# Supplementary material for: Fatty Acids and Metabolomic Composition of Follicular Fluid Collected from Environments Associated with Good and Poor Oocyte Competence in Goats
Source: Int J Mol Sci. 2022 Apr 8;23(8):4141. doi: 10.3390/ijms23084141 (PMC9028732; doi:10.3390/ijms23084141)
Supplement: Supplementary file 1 [file ijms-23-04141-s001.zip › ijms-1658295-supplementary.pdf]

International Journal of *Molecular Sciences*

MDPI

Supplementary Materials:

Table S1: Metabolomic study of FF of adult and prepubertal individuals. Results of the statistical significance tests performed on the buckets according to Goodpaster et al. [19]. First 50 buckets ordered by its p-value are shown.

Significant buckets if p < 0.0002451 (Bonferroni corrected confidence interval)
final significance value obtained from combined tests

Buckets rrdered by p-value

ppm	p-value	Shapiro-1	Shapiro-2	Kruskal-Wallis	Welch T-Test
4.125	4.002499E-19	8.054231E-01	6.958099E-02	9.968593E-09	4.002499E-19
1.325	1.842807E-15	8.174504E-02	7.031185E-01	7.222255E-09	1.842807E-15
3.725	6.482473E-09	6.752399E-02	3.144754E-02	6.482473E-09	2.690525E-10
3.475	3.515282E-08	8.767856E-03	3.344762E-03	3.515282E-08	1.120473E-08
3.225	6.488145E-08	1.038217E-01	3.556596E-02	6.488145E-08	2.239308E-11
5.275	2.592651E-07	1.142751E-02	2.473802E-02	2.592651E-07	1.982318E-07
4.075	2.592651E-07	5.426523E-02	7.153976E-03	2.592651E-07	5.385819E-09
5.325	6.111841E-07	9.632843E-03	7.859115E-02	6.111841E-07	3.246501E-07
3.875	7.369079E-07	4.945781E-03	9.184126E-02	7.369079E-07	2.803619E-06
4.675	8.873643E-07	1.816263E-03	1.654227E-01	8.873643E-07	5.383263E-06
1.575	2.634184E-06	2.521762E-01	5.851686E-06	2.634184E-06	8.570567E-04
3.825	3.001516E-06	1.594913E-01	1.779204E-01	2.634184E-06	3.001516E-06
1.625	6.299200E-06	1.538006E-01	6.276157E-03	6.299200E-06	4.146477E-05
1.175	2.024486E-05	6.745527E-02	8.578043E-04	2.024486E-05	3.050195E-04
3.425	2.379886E-05	2.234517E-03	6.231569E-01	2.379886E-05	1.507159E-05
1.075	2.766644E-05	3.912226E-01	9.917117E-01	4.488021E-05	2.766644E-05
1.125	3.687568E-05	3.847647E-01	1.975878E-01	6.116872E-05	3.687568E-05
2.775	6.116872E-05	2.136022E-03	6.910448E-01	6.116872E-05	1.948282E-04
0.775	6.396748E-05	1.257613E-01	9.348096E-01	7.690506E-05	6.396748E-05
0.925	8.295156E-05	4.323272E-03	8.529402E-01	8.295156E-05	5.937280E-05
2.725	9.641748E-05	3.823315E-03	5.363636E-01	9.641748E-05	2.575167E-04
2.675	1.039005E-04	1.052404E-03	9.665702E-01	1.039005E-04	2.074961E-04
2.375	1.133134E-04	1.496881E-01	3.114339E-01	1.459454E-05	1.133134E-04
3.925	1.502757E-04	9.752648E-02	1.305492E-02	1.502757E-04	6.880533E-04
5.375	1.868201E-04	2.496464E-03	2.074212E-01	1.868201E-04	3.088355E-04
1.675	1.925219E-04	1.276121E-01	7.311299E-01	1.616352E-04	1.925219E-04
2.625	2.486416E-04	1.412293E-03	5.113706E-01	2.486416E-04	4.012447E-04
1.025	2.709851E-04	7.191152E-02	7.852262E-01	2.486416E-04	2.709851E-04
3.525	2.853308E-04	1.274688E-01	6.200930E-01	1.600467E-03	2.853308E-04
7.075	4.971524E-04	4.899418E-02	2.012636E-03	4.971524E-04	1.436224E-03
7.175	5.689247E-04	9.421538E-03	8.366702E-03	5.689247E-04	1.411768E-03
7.125	5.689247E-04	2.473840E-02	6.279179E-04	5.689247E-04	1.353206E-03
5.125	6.502522E-04	1.586942E-03	1.108699E-01	6.502522E-04	1.362712E-03
0.625	6.768678E-04	7.069336E-01	1.085073E-01	4.051646E-04	6.768678E-04
7.025	6.948543E-04	2.162670E-02	2.235528E-02	6.948543E-04	2.000877E-03
3.325	6.948543E-04	4.900612E-04	6.795304E-02	6.948543E-04	6.159497E-04
6.975	7.422860E-04	7.662018E-02	1.384665E-02	7.422860E-04	2.034697E-03
3.275	7.644849E-04	9.109092E-01	6.280984E-01	5.689247E-04	7.644849E-04
0.725	8.362982E-04	5.161254E-01	9.918299E-01	6.502522E-04	8.362982E-04
5.175	8.462984E-04	1.242023E-03	2.232359E-01	8.462984E-04	1.305743E-03
7.375	9.032301E-04	8.479272E-01	8.491227E-03	9.032301E-04	8.261710E-04
5.075	9.032301E-04	1.844472E-03	7.791920E-02	9.032301E-04	1.539453E-03
5.925	9.636941E-04	3.734775E-03	3.162586E-01	9.636941E-04	2.551047E-03
5.425	1.503751E-03	1.361796E-03	2.570918E-01	1.503751E-03	1.910437E-03
1.725	1.521416E-03	1.172916E-01	9.511483E-01	1.244981E-03	1.521416E-03
7.325	1.600467E-03	1.872222E-02	8.180614E-04	1.600467E-03	1.987858E-03
7.225	1.702879E-03	1.034737E-03	4.270379E-03	1.702879E-03	2.371547E-03
3.175	1.811288E-03	1.420157E-01	3.665494E-02	1.811288E-03	2.279316E-03
5.475	1.926008E-03	1.231272E-03	3.306904E-01	1.926008E-03	2.448180E-03
2.475	1.926008E-03	1.503060E-02	7.814143E-01	1.926008E-03	4.604114E-03

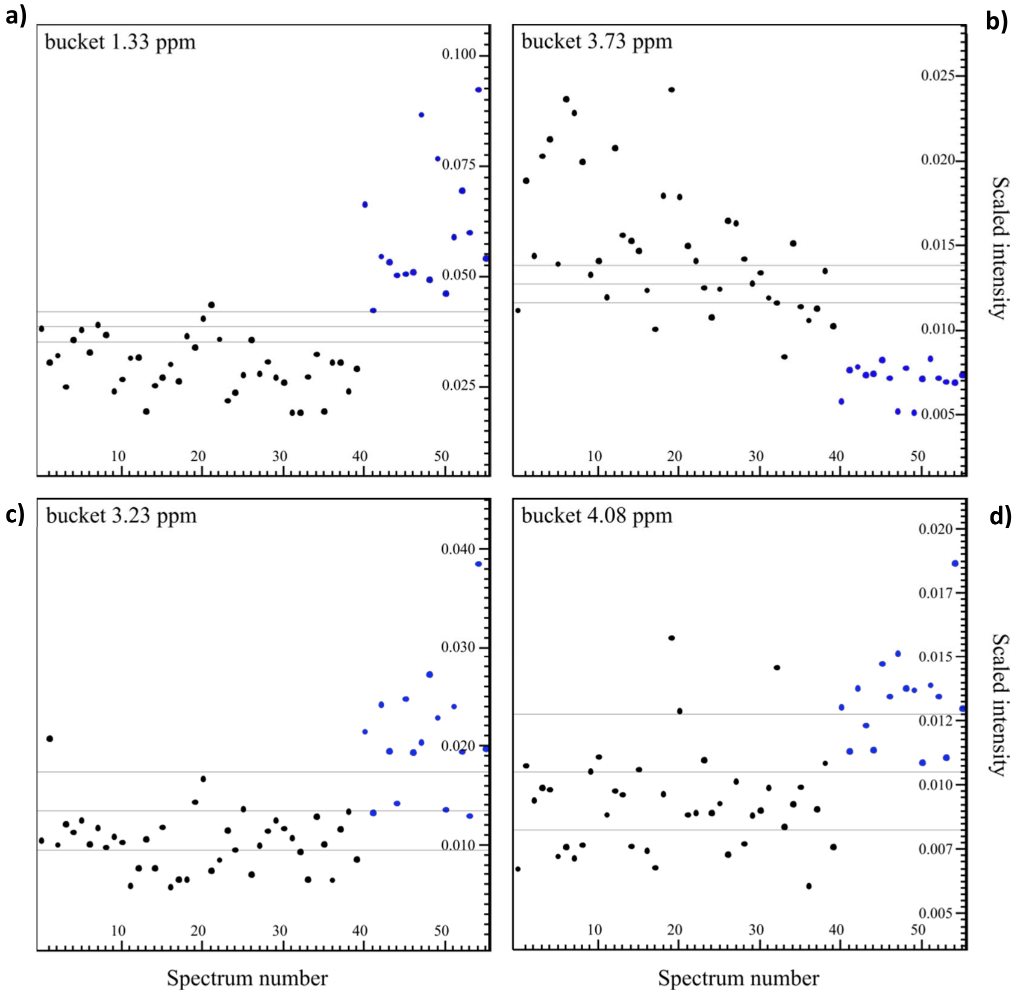

Figure S1: Metabolomic study of FF of adult and prepubertal individuals. Plots showing the distribution of normalized data points for some discriminant buckets assigned to lactate, glucose, -N(CH3)3 groups and inositol. Prepubertal and adult samples are indicated as blue and black dots, respectively. a) 1.33 ppm bucket, P = 1.84 x 10-15, lactate; b) 3.73 ppm bucket, P = 6.48 x 10-9, glucose; c) 3.23 ppm bucket, P = 6.49 x 10-8, -N(CH3)3 groups; d) 4.08 ppm bucket, P = 2.59 x 10-7, inositol.

Table S2: Metabolomic study of FF of prepubertal individuals. Results of the statistical significance tests performed on the buckets according to Goodpaster et al. [19]. First 50 buckets ordered by p-value are shown.

Significant buckets if p < 0.00035971 (Bonferroni corrected confidence interval)
final significance value obtained from combined tests

Buckets ordered by p-value

ppm	p-value	Shapiro-1	Shapiro-2	Kruskal-Wallis	Welch T-Test
**3.525**	1.353721E-06	7.567722E-01	4.331919E-01	1.836791E-03	1.353721E-06
**3.625**	1.074592E-05	1.796609E-01	8.241885E-01	1.836791E-03	1.074592E-05
**3.775**	2.426175E-05	2.039727E-01	2.768875E-01	1.836791E-03	2.426175E-05
3.575	1.836791E-03	2.936128E-02	5.685548E-01	1.836791E-03	1.704962E-06
3.225	1.836791E-03	2.701554E-02	8.953341E-02	1.836791E-03	1.144969E-03
3.275	4.906314E-03	4.777517E-01	9.923037E-01	7.761477E-03	4.906314E-03
4.075	7.761477E-03	1.781863E-02	8.069055E-01	7.761477E-03	1.656407E-02
7.225	1.486313E-02	6.199574E-01	5.414660E-03	1.486313E-02	1.086622E-01
2.175	2.651161E-02	5.734149E-01	6.936293E-02	2.716625E-02	2.651161E-02
3.725	2.716625E-02	5.436315E-01	1.241446E-02	2.716625E-02	3.414307E-02
0.075	2.716625E-02	9.611192E-01	1.088461E-02	2.716625E-02	4.489382E-02
1.825	3.609706E-02	3.158434E-02	3.917399E-01	3.609706E-02	6.341036E-02
1.775	3.609706E-02	1.714956E-02	2.666314E-01	3.609706E-02	8.973938E-02
1.525	3.609706E-02	1.355632E-02	1.092053E-01	3.609706E-02	6.723201E-02
1.075	3.609706E-02	2.815415E-02	5.944706E-02	3.609706E-02	1.135281E-01
1.025	3.609706E-02	1.267499E-02	3.392853E-02	3.609706E-02	2.145387E-01
7.175	4.741837E-02	4.982636E-02	7.771520E-02	4.741837E-02	1.084564E-01
7.075	4.741837E-02	1.743024E-02	3.556711E-01	4.741837E-02	9.577874E-02
1.975	4.741837E-02	1.664918E-02	5.245320E-02	4.741837E-02	2.345135E-01
1.725	4.741837E-02	6.767584E-03	1.143042E-01	4.741837E-02	1.745402E-01
1.675	4.741837E-02	9.244285E-03	2.697616E-01	4.741837E-02	2.065591E-01
1.625	4.741837E-02	8.900012E-03	3.602768E-01	4.741837E-02	1.896848E-01
1.425	4.741837E-02	8.284819E-03	1.037022E-01	4.741837E-02	2.145951E-01
1.925	6.158722E-02	1.719780E-02	1.259176E-01	6.158722E-02	2.201323E-01
1.575	6.158722E-02	9.700423E-03	2.623388E-01	6.158722E-02	2.164215E-01
1.475	6.158722E-02	6.156996E-03	2.632594E-02	6.158722E-02	5.630261E-01
1.875	6.293240E-02	5.409981E-02	5.236255E-01	2.716625E-02	6.293240E-02
5.925	7.909393E-02	5.006453E-03	3.919968E-02	7.909393E-02	7.392818E-01
4.425	7.909393E-02	8.232175E-01	1.142556E-02	7.909393E-02	4.999103E-01
0.975	7.909393E-02	9.510544E-03	3.434716E-02	7.909393E-02	4.932351E-01
-0.125	7.909393E-02	4.334358E-02	5.284944E-02	7.909393E-02	3.711588E-01
-0.175	7.909393E-02	3.019816E-02	4.694970E-02	7.909393E-02	3.873199E-01
-0.225	7.909393E-02	2.263163E-02	3.947594E-02	7.909393E-02	3.997535E-01
-0.275	7.909393E-02	1.844059E-02	3.529825E-02	7.909393E-02	4.157735E-01
-0.325	1.004493E-01	1.563111E-02	3.154525E-02	1.004493E-01	4.392916E-01
2.425	1.144499E-01	6.238587E-01	1.148291E-01	7.909393E-02	1.144499E-01
7.125	1.261675E-01	1.895638E-02	9.867259E-02	1.261675E-01	1.626950E-01
4.475	1.261675E-01	4.555508E-01	4.560725E-03	1.261675E-01	6.260735E-01
4.375	1.261675E-01	7.252204E-01	8.393282E-03	1.261675E-01	5.587738E-01
3.375	1.261675E-01	6.029721E-02	6.727587E-03	1.261675E-01	3.990530E-01
2.525	1.261675E-01	9.493045E-02	4.054950E-03	1.261675E-01	5.406583E-01
7.025	1.512867E-01	7.881179E-02	7.749002E-02	1.567454E-01	1.512867E-01
7.325	1.530689E-01	9.014356E-01	5.385313E-02	1.567454E-01	1.530689E-01
1.125	1.709025E-01	1.145555E-01	1.234673E-01	4.741837E-02	1.709025E-01
4.225	1.779353E-01	7.035288E-01	6.549637E-02	4.741837E-02	1.779353E-01
6.975	1.926380E-01	2.733686E-02	1.266488E-01	1.926380E-01	2.216969E-01
5.325	1.926380E-01	5.731142E-01	4.417154E-02	1.926380E-01	2.359885E-01
2.575	1.926380E-01	6.736064E-02	6.921479E-03	1.926380E-01	6.718404E-01
1.375	1.926380E-01	4.823044E-02	1.335560E-01	1.926380E-01	5.189107E-01
2.225	2.114987E-01	6.955285E-01	7.013311E-01	1.926380E-01	2.114987E-01

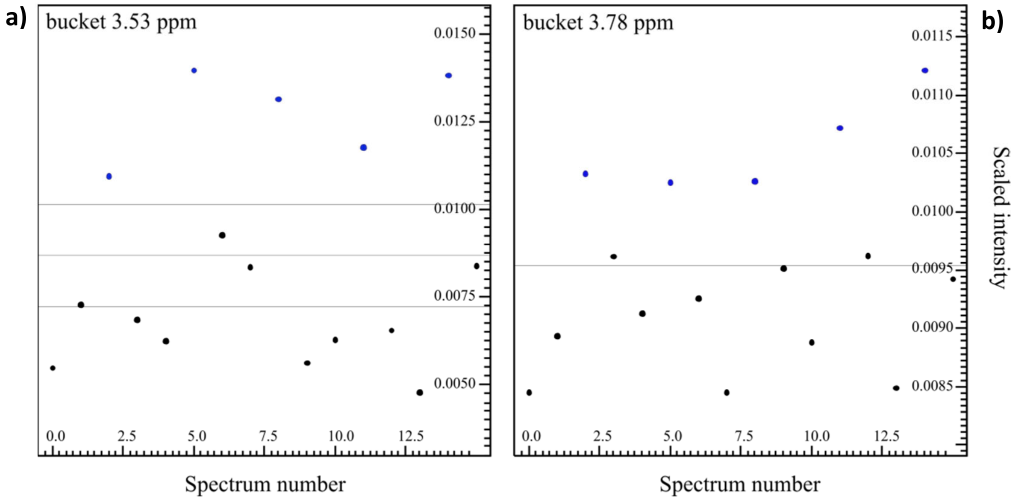

Figure S2: Metabolomic study of FF of prepubertal individuals. Plots showing the distribution of normalized data points for some discriminant buckets assigned to inositol and lysine. Samples with small and large follicles are indicated as blue and black dots, respectively. a) 3.53 ppm bucket, P = 1.35 x 10^{-6}, inositol; b) 3.78 ppm bucket, P = 2.43 x 10^{-5}, lysine.
